# An Updated Review of Porcine Deltacoronavirus in Terms of Prevalence, Pathogenicity, Pathogenesis and Antiviral Strategy

**DOI:** 10.3389/fvets.2021.811187

**Published:** 2022-01-13

**Authors:** Cong Duan

**Affiliations:** China Institute of Veterinary Drug Control, Beijing, China

**Keywords:** porcine deltacoronavirus, prevalence, pathogenicity, pathogenesis, antiviral strategy

## Abstract

The recent experience with SARS-COV-2 has raised our alarm about the cross-species transmissibility of coronaviruses and the emergence of new coronaviruses. Knowledge of this family of viruses needs to be constantly updated. Porcine *deltacoronavirus* (PDCoV), a newly emerging member of the genus *Deltacoronavirus* in the family *Coronaviridae*, is a swine enteropathogen that causes diarrhea in pigs and may lead to death in severe cases. Since PDCoV diarrhea first broke out in the United States in early 2014, PDCoV has been detected in many countries, such as South Korea, Japan and China. More importantly, PDCoV can also infect species other than pigs, and infections have even been reported in children, highlighting its potential for cross-species transmission. A thorough and systematic knowledge of the epidemiology and pathogenesis of PDCoV will not only help us control PDCoV infection, but also enable us to discover the common cellular pathways and key factors of coronaviruses. In this review, we summarize the current knowledge on the prevalence, pathogenicity and infection dynamics, pathogenesis and immune evasion strategies of PDCoV. The existing anti-PDCoV strategies and corresponding mechanisms of PDCoV infection are also introduced, aiming to provide suggestions for the prevention and treatment of PDCoV and zoonotic diseases.

## Introduction

Porcine deltacoronavirus (PDCoV) is one of the most common enteropathogenic virus in the swine industry, causing typical clinical symptoms characterized by acute diarrhea, vomiting, dehydration and even death in piglets (1, 2). It belongs to the genus *Deltacoronavirus* in the family *Coronaviridae* of the order Nidovirales (3). It's an enveloped virus possessing a single-stranded positive-sense RNA genome of approximately 25.4 kb in size. The PDCoV genome arrangements are as follows: 5' untranslated region (UTR), open reading frame 1a/1b (ORF1a/1b), spike (S), envelope (E), membrane (M), non-structural protein 6 (NS6), nucleocapsid (N), NS7, NS7a and 3' UTR (4). ORF1a and ORF1b encompass the 5'-proximal two-thirds of the genome and encode two viral replicase precursor polyproteins, pp1a and pp1ab, which are predicted to be processed into non-structural proteins responsible for viral replication and transcription. The 3'-proximal last third of the genome codes for the four structural proteins (S, E, M and N), as well as at least three accessory proteins [NS6 (between the M and N genes), NS7 (within the N gene) and NS7a (within the N gene)] (5–7). A recent study found that PDCoV NS6 protein is expressed during infection *in vivo* and incorporated into PDCoV virions, inferring that NS6 protein is not only a PDCoV accessory protein but also a component associated with PDCoV virions (8).

PDCoV was first reported to be detected in pig rectal swabs in Hong Kong in 2012, when molecular surveillance studies were conducted to examine the diversity of coronaviruses in mammals and birds (9). PDCoV diarrhea broke out in the USA for the first time in early 2014 (3). Until now, PDCoV has been detected in many countries, such as South Korea, Japan and China (10–12). Moreover, experimental infection studies confirmed that calves, chickens, turkey poults, mice are susceptible to infection with PDCoV, and independent infections of PDCoV among Haitian children have been reported (13–17). Therefore, it is necessary to develop effective measures to prevent and control PDCoV.

A comprehensive understanding of the epidemiology and pathogenesis of PDCoV conduce to prevent PDCoV infection. This review focuses on the current knowledge on the prevalence, pathogenicity and infection dynamics, pathogenesis and immune evasion strategies of PDCoV, as well as existing methods of preventing and treating PDCoV and their corresponding mechanisms.

## Prevalence of PDCoV

Deltacoronaviruse in pigs was discovered for the first time during the molecular surveillance study, named porcine coronavirus HKU15 (9). PDCoV diarrhea broke out for the first time in Ohio, USA in early 2014 (3). Soon, swine samples from farms in nine other USA States (Minnesota, South Dakota, Nebraska, Illinois, Indiana, Michigan, Kentucky, Pennsylvania, and Iowa) were also tested positive for PDCoV (4, 18). Until now, PDCoV has been detected in multiple countries, including Canada, China, South Korea, Thailand, Vietnam, Lao PDR, Japan, Mexico and Peru (10, 11, 19–23). Retrospective researches revealed the presence of PDCoV in pigs before its first report in 2012. For example, Thachil et al. detected PDCoV IgG antibodies in serum samples collected in 2010 from USA farms (24). Dong et al. also found that two samples collected in 2004 from Anhui, China were positive for PDCoV (25). The majority of PDCoVs could be divided into four lineages based on the full genomes: the Thailand, Early China, USA, and China (26). The Thailand lineage contains strains from Vietnam, Laos, and Thailand. The Early China and China lineages contain strains only from China including CHN-AH-2004 (GenBank accession no. KP757890). The USA lineage contains strains from the USA, Japan, Korea, Mexico, Peru and China [AH2019/H (GenBank accession no. MN520198) and SD2019/426 (GenBank accession no. MN520191)] (22, 23, 26). Both the USA and China lineages are the major prevailing genotypes worldwide (26). The China lineage shows the largest genetic divergence, followed by the Thailand lineage, and the USA lineage indicates the least genetic divergence (27). More frequent intra- and inter-lineage recombination and higher virus genetic diversity in the China lineages compared with the USA lineage (26). In the China and Thailand lineages, recombination occurs frequently within ORF1ab, while strains from the USA lineage do not exhibit recombination within the full-length genome (26). According to existing studies, the positive rates of PDCoV in clinical samples of diarrheal pigs in different periods and regions are different, as shown in the Table 1.

**Table 1:** The positive rate of PDCoV in clinical samples of diarrheal pigs in different periods and regions.

**Period**	**Region**	**Samples**	**Positive**	**Positive rate (%)**	**Coinfection of PDCoV/PEDV**	**Positive rate^a^ (%)**	**Reference**
February–April, 2014	10 states in the USA (Minnesota, South Dakota, Nebraska, Illinois, Indiana, Michigan, Kentucky, Pennsylvania, Maryland and Ohio)	435	109	25	19	4.37	(18)
January–February, 2014	7 states in the USA (Ohio, Michigan, Illinois, Minnesota, Nebraska, South Dakota and Missouri) and Canada	293	89	30	29	9.90	(28)
November 2012–March 2015	Jiangxi, China	356	120	33.71	70	19.67	(20)
2004–2014	4 provinces in China (Anhui, Guangxi, Hubei and Jiangsu)	215	14	6.51	7	3.26	(25)
2012–2014, July–August 2015	3 provinces in China (Guangdong, Hainan and Guangxi)	390	5	1.28	5	1.28	(29)
January 2014–December 2016	South Korea	683	130	19.03	43	6.3	(10)
2015	Thailand, Vietnam, Lao PDR and Philippines	97	12	12.4	12	12.4	(21)
December 2015–June 2016	Guangdong, China	252	55	21.8	2	0.79	(30)
November 2013–August 2014	Japan	477	72	15.1	0	0	(11)
January 2016–May 2017	Taiwan, China	172	29	16.9	14	8.1	(31)
April 2015–March 2018	Henan, China	430	101	23.49	61	14.19	(32)
2012–2018	5 provinces in China (Jiangxi, Zhejiang, Fujian, Guangdong and Hunan)	2987	813	27.22	380	12.72	(33)
2014–2017	Mexico	885	85	9.6	46	5.2	(22)
2011–2016	Vietnam	108	11	10.19	11	10.19	(34)
March 2016–June 2018	18 provinces in China (Heilongjiang, Liaoning, Beijing, Hebei, Shanxi, Shandong, Henan, Jiangsu, Anhui, Hubei, Zhejiang, Jiangxi, Fujian, Hunan, Guangxi, Yunnan, Sichuan, Gansu)	719	94	13.07	34	4.73	(27)
January 2017–June 2019	Sichuan, China	634	84	13.25	47	7.41	(35)

a *Positive rate, Positive rate of PDCoV-PEDV coinfection*.

## Pathogenicity and Infection Dynamics of PDCoV

### Clinical Manifestation of PDCoV Infection

PDCoV can infect pigs of different ages, among which piglets are more susceptible (3, 12, 20, 35–39). The typical clinical symptoms of PDCoV infection are characterized by acute and severe watery diarrhea, vomiting, and dehydration, sometimes accompanied by lethargy and anorexia (1, 12, 36, 40, 41). Generally, clinical signs develop at 1–2 days post-inoculation (dpi), and the progression of diarrhea is most severe at 3–7 dpi and the pigs gradually recover thereafter (12, 36, 40–42).

### Gross Lesions and Histological Lesions of PDCoV Infection

Currently, a majority of studies focus on necropsy of PDCoV-inoculated pigs at 3–7 dpi. Gross lesions mainly appear in the gastrointestinal tract, especially the small intestine, and are characterized by thin and transparent intestinal walls, accumulation of a large amount of yellow fluid in the intestinal lumen, and occasionally, coagulated milk in the stomach (12, 36, 40, 41, 43). One study reported that the infected pigs euthanized at 23–24 or 28 dpi showed no gross lesions (36).

Histological lesions are primarily observed in the jejunum and ileum. The typical histological lesions include multifocal to diffuse villous enterocyte swelling and vacuolation, and moderate to severe villous blunting and atrophy (12, 36, 39, 41, 42, 44). Villous enterocytes are mildly to moderately attenuated with sloughing enterocytes into the lumen. The villus height-to-crypt depth ratio decreases significantly. Occasionally, the lamina propria of the small intestine is hyperemic, or a small number of lymphocytes and neutrophils infiltrate the lamina propria (11, 32–35). Several studies also found histologic lesions in the stomach, cecum, colon and lung (1, 36, 40). At 10–11 dpi, the intestinal tract exhibits varying degrees of recovery (39, 42).

### Virus Distribution in PDCoV-Inoculated Pigs

As mentioned above, most of the current studies euthanize PDCoV-inoculated pigs at 3–7 dpi. Based on the above research conditions, PDCoV antigens were observed mainly in the small intestine, and the jejunum and ileum had more PDCoV antigen-positive enterocytes compared with the duodenum (12, 36, 40, 42–44). Meanwhile, PDCoV antigens were generally observed in the cytoplasm of villous epithelial cells, but infrequently in crypt epithelial cells (12, 36, 40, 42–44). PDCoV mainly aggregates in the duodenum in the early stage, while in the peak of stage and the later period, the virus transfers to ileum (42). Occasionally, a small number of PDCoV antigen-positive cells were detected in the cecum and colon (36, 39), and fewer PDCoV antigen-positive cells were detected in mesenteric lymph nodes (MLN) (36), peyer's patches (39) and stomach (45). One study reported that the percentage of pigs that tested positive for PDCoV antigen decreased significantly at 7–8 dpi, and no positive cells were detected in samples from 10 dpi and thereafter (39). PDCoV antigens were not observed in other organs, such as heart, liver, spleen, lung, kidney and tonsil. However, different levels of PDCoV RNA were detected in multiple organs by RT-PCR. Although the strain and dose of the virus, the age of pigs or the time of euthanasia varied in different studies, PDCoV RNA was usually detected in the duodenum, jejunum, ileum, cecum, colon, liver, spleen, kidney, lung and MLN (2, 12, 39–41, 45), and the quantities are relatively high in the duodenum, jejunum, ileum, cecum and colon. Low to moderate quantities of viral RNA are occasionally detected in the rectum, stomach, diaphragm, heart, inguinal lymph node, tonsil and muscle from rear leg (41, 45). One study using 5-day-old piglets orally inoculated with PDCoV (CHN-GD16–05) with 5 mL at 10^8.6^ TCID_50_/mL declared that viral RNA was detected from duodenum, jejunum, and ileum at the early stage. The small intestine had the highest viral copies in the middle stage. Afterwards, the virus could be detected in low quantities in ileums during the late stage while other tissue samples were negative (42). Vitosh-Sillman reported that most of the lymph node and small intestinal samples from pigs were moderately positive from day 6 to the end of the study at day 42 (39).

PDCoV infection can cause viremia (40). Some studies have detected PDCoV RNA in relatively low amounts in the serum or blood (40, 41), while others have not (1, 12). For example, Chen et al. inoculated 5-day-old piglets with the PDCoV cell culture isolate with the titer of 3 × 10^3^ TCID_50_/mL (10 mL per pig). At 3 dpi, viral RNA was detected in 2 of 8 PDCoV inoculated pigs with the titer of 10^2.2^-10^2.9^ TCID_50_/mL. From 4 to 7 dpi, viral RNA was detected in serum of all pigs with the titer ranges of 10^0.5^-10^3^ TCID_50_/mL (41). Whereas, in the study performed by Jung et al. no PDCoV-inoculated pigs at 72–168 h post-inoculation (hpi) had detectable virus RNA in serum (1).

### Fecal Viral Shedding and Specimen to Monitor Swine Herd Health

Based on experimental findings, fecal viral shedding occurs on 24–48 hpi, peaks at 2–7 dpi (12, 40, 41, 44, 45). Fecal viral RNA is still detectable at 14–35 dpi (12, 40). In addition to feces and tissue samples (especially intestines), oral fluids and environmental samples are also utilized to monitor or detect PDCoV. Evaluation of the association between the occurrence and specimen of PDCoV in diagnostic samples uncovers that oral fluids have the highest relative odds associated with PDCoV detection, indicating that oral fluids are useful for determining whether a swine herd is infected with PDCoV, and are a valuable tool for monitoring the health of swine herd. Besides, the relative odds of PDCoV detection in feces are higher than in fecal swabs and intestines (46).

### PDCoV Infection in Species Other Than Pigs

Experimental infection studies show that calves, chickens, turkey poults, mice are susceptible to infection with PDCoV. PDCoV-inoculated chickens and turkey poults exhibit diarrhea, shed detectable viral RNA from the cloaca and trachea and have distended gastrointestinal tracts containing a mixture of yellow liquid and gas (15). Histological lesions are observed in the lung, kidney, and intestinal tissues of infected chickens (14, 15). Besides, Boley et al. assigned chickens or turkey poults from each uninfected group and allowed them to comingle with the infected group, and they found that PDCoV spread rapidly from infected to naive chickens or turkey poults (15). PDCoV-inoculated calves have prolonged viral RNA shedding, but show no clinical signs or obvious intestinal lesions, implying that the infectivity of PDCoV in calves is limited (13). PDCoV is also able to limitedly infect mice (16). PDCoV RNA is mainly detected in the intestine and lymphoid tissues of mice, while no similar symptoms are observed. Such asymptomatic infection may support these species as reservoirs of PDCoV to participate in further interspecific transmission (16). Moreover, PDCoV is identified in plasma samples of three Haitian children with acute undifferentiated febrile illness, and Vero E6 cells inoculated with plasma from one patient show non-specific CPE at 11 dpi (17).

## Pathogenesis of PDCoV

### Receptor Binding and Cellular Entry

LLC-PK cells in general are more susceptible to PDCoV infection than ST cells (47, 48). One replication cycle of PDCoV takes 5–6 h (49). Both PDCoV genomes and viral titers reach a peak at 24 hpi, after which a plateau is reached (50). The prerequisite of coronavirus infection is its entrance into the host cell. Coronavirus entry is a multi-step process involving viral attachment and fusion of viral and cellular membranes, which relies on the interaction between coronavirus S proteins and specific receptors on host cell surfaces. PDCoV S protein is a type I transmembrane glycoprotein that contains three receptor-binding S1 subunits and three membrane fusion S2 subunits (51, 52). PDCoV receptor has not been clearly elucidated, and there are many controversies about whether aminopeptidase N (APN, also called CD13) is the receptor for PDCoV (16, 53–57). Transmissible gastroenteritis virus (TGEV) employs APN as its receptor (58).

After binding to the receptor, conformational changes occur between S1 and S2, exposing the cleavage site to proteases. The host protease or exogenous protease cleaves the spike, releasing the spike fusion peptide and facilitating virus entry. Zhang et al. reported that cathepsin L and cathepsin B in lysosomes and exogenous trypsin in cell cultures independently induce PDCoV S protein cleavage and fusion, uncovering two distinct PDCoV entry pathways: one through cathepsin L and cathepsin B in the endosome and another via a protease at the cell surface. Trypsin activates membrane fusion induced by S protein in ST cells, and Arg-672 in the S protein is critical for trypsin-induced cell fusion (59). Yang et al. confirmed that trypsin does not affect PDCoV entry into LLC-PK cells and ST cells, but enhances cell-to-cell fusion in a cell type-dependent manner at a late stage of the viral infection to promote PDCoV replication (47). PDCoV enters IPI-2I cells via macropinocytosis independent of specific receptors and clathrin-mediated endocytosis dependent on a low-pH environment and dynamin rather than caveola-mediated endocytosis (60). The endocytotic markers Rab5 and Rab7, but not Rab11, regulated PDCoV endocytosis. Nonetheless, the caveola-mediated endocytosis is utilized by PDCoV to enter ST cells and LLC-PK1 cells. APN and PDCoV virions are co-localized with Rab5, Rab7, and LAMP1, implying that APN mediates PDCoV entry by an endocytotic pathway (61). No matter which receptor engages, only entry by an endocytotic route ultimately leads to efficient viral replication. Additionally, the cholesterol in the cell membrane and viral envelope is a key component for viral entry (62).

### Cellular Factors Involved in PDCoV Infection

Effective viral infection depends on the host cell machinery. Virus orchestrates numerous cellular processes to benefit its replication. Mitogen-activated protein kinase (MAPK) signaling pathway converts extracellular stimuli into a wide range of cellular responses. Extracellular signal-regulated kinase (ERK, also known as p42/44 MAPK), Janus kinase (JNK, also known as stress activated protein kinase-1, SAPK1) and p38 MAP kinase (also known as SAPK2/RK) are three major MAPK signaling pathways in mammals. Viruses utilize MAPK signaling pathways to manipulate cellular functions for their own benefit. Pharmacologic inhibition of MAPK signaling pathways exhibits antiviral efficacy, representing a potential therapy. PDCoV activates the ERK signaling pathway to promote its replication and viral attachment is responsible for monophasic ERK activation (63). Pharmacological inhibition of ERK activation has no effect on viral entry but impairs the post-entry steps of PDCoV replication cycle, including viral RNA synthesis, viral protein translation, and viral progeny production. ERK1/2 activation is irrelevant to PDCoV-induced apoptosis. Recently, our *in vitro* study found that PDCoV activates p38 signaling pathway to favor its replication (64). Later, we employed piglet models and illustrated that phosphorylated p38 levels in jejunum and ileum of PDCoV-infected piglets upregulated (2). Jeon et al. confirmed that JNK1/2 and p38 were involved in the replication of PDCoV by affecting viral biosynthesis and progeny release (65). Remarkably, pharmacological inhibition of JNK1/2 or p38 activation alters the synthesis of cytokines during PDCoV infection. For example, the mRNA levels of IL-1α, TNF-α and IFN-β were decreased, while the mRNA levels of IL-13, IL-7 and IFN-γ were increased, highlighting the regulatory effect of JNK1/2 and p38 signaling pathway on immune response during PDCoV infection.

NF-κB signaling pathway is a key mediator of cytokines and plays a central role in the host response to viral infection. PDCoV infection activates the NF-κB signaling pathway *in vitro* and *in vivo* (2, 64). However, persistent activation of NF-κB signaling pathway can trigger inflammation by stimulating the expression of vast cytokines.

When faced with viral infection, host cells use autophagy to transport viruses to the lysosomal compartment for degradation and elimination. Some viruses have evolved multiple strategies to hijack autophagy in favor to replication. Our recent study using LLC-PK1 cell models and piglet models of PDCoV infection reveals that PDCoV-induced autophagy facilitates virus replication, and the p38 signaling pathway mediates this process (66). However, which protein of PDCoV is the key inducer of autophagy has not been identified.

The host can eliminate virus-infected cells through apoptosis. However, some viruses trigger apoptosis to promote dissemination of viral progeny and cause cytopathic effect *in vitro* and/or tissue damage *in vivo*. PDCoV infection stimulates mitochondrial outer membrane permeabilization either via Bax recruitment or mitochondrial permeability transition pore opening to permit the release of apoptogenic mitochondrial cytochrome c into the cytoplasm, thereby leading to the caspase-dependent intrinsic apoptosis to facilitate viral replication (67). Our study reveals that PDCoV infection induces apoptosis in both LLC-PK1 cells and piglet intestines, and the p38 signaling pathway is involved in PDCoV-induced caspase-dependent apoptosis (2, 64). However, Jung et al. reports that PDCoV induces apoptosis in ST and LLC-PK1 cells *in vitro* but not in infected intestinal enterocytes *in vivo* (68). Piglet age and necropsy time may be responsible for this discrepancy, and further investigation need to be carried out.

Ionic calcium (Ca^2+^) is a versatile intracellular second messenger. PDCoV modulates calcium influx to facilitate replication. The Ca^2+^ channel blocker diltiazem hydrochloride inhibits PDCoV infection targeting the replication step of the viral replication cycle. Knockdown of the L-type Ca^2+^ voltage-gated channel subunit CACNA1S also restricts PDCoV replication (69). Cholesterol 25-hydroxylase (CH25H), a key enzyme regulating cholesterol metabolism, is regarded as a broad host antiviral factor. PDCoV infection remarkably increases the expression of CH25H in IPI-FX cells, and CH25H plays a negative role in PDCoV infection in a manner that is not entirely dependent on its enzyme activity (70). Additionally, proteomic analysis of cells indicates that PDCoV upregulated proteins are enriched in PI3K-AKT signaling pathway, and downregulated proteins are enriched in the mTOR signaling pathway. PI3K-AKT-mTOR signaling pathway plays an important role in many physiological and pathological conditions, and may be key adaptor molecules in inhibiting viral replication (71).

### Immune Evasion of PDCoV

**Figure 1:**
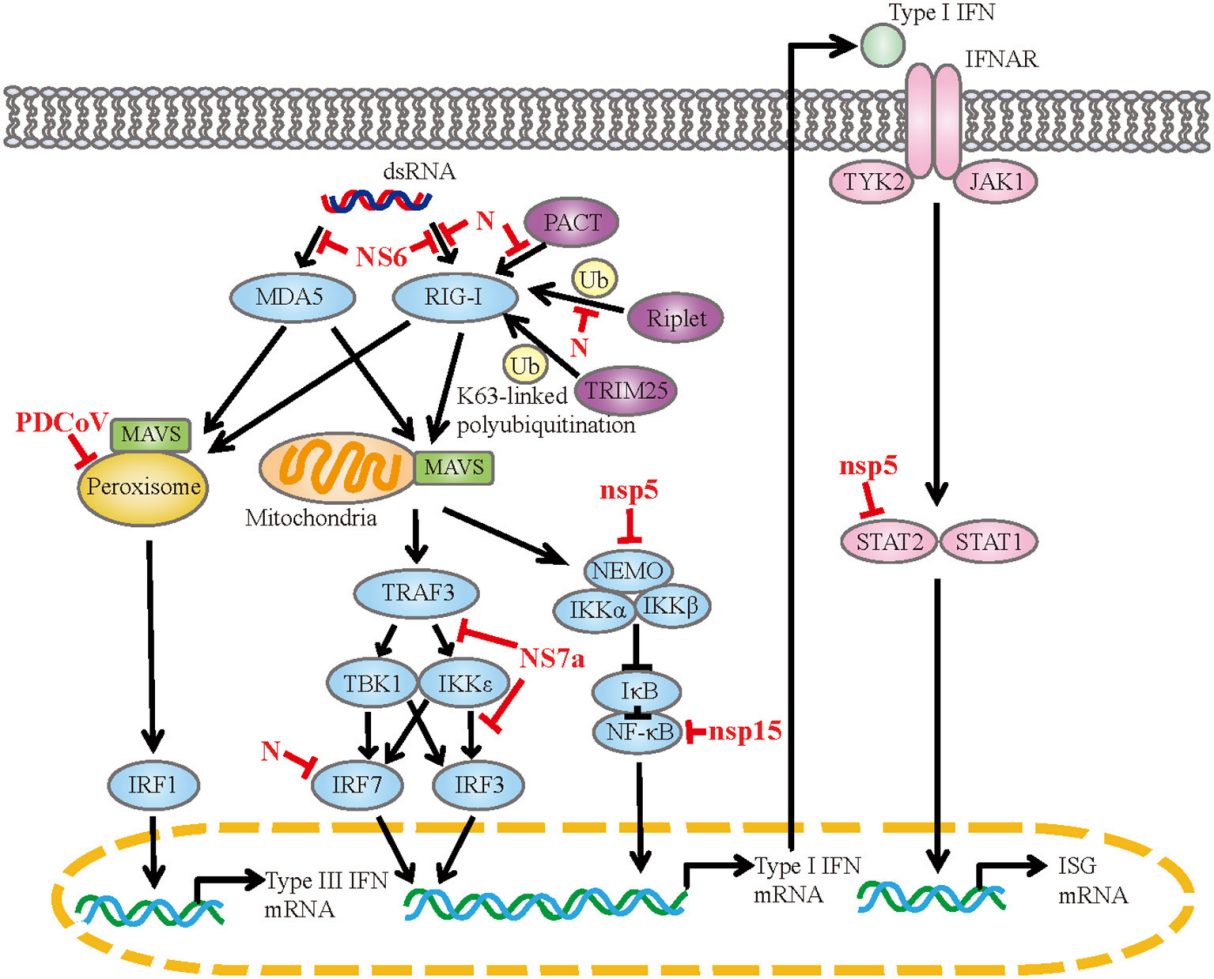
The mechanism of PDCoV interfered with IFN response. dsRNA, double-strand RNA; RIG-I, retinoic acid-inducible gene I; MDA5, melanoma differentiation-associated protein 5; PACT, protein activator of protein kinase R; TRIM25, tripartite motif containing 25; MAVS, mitochondrial antiviral signaling protein; TRAF3, tumor necrosis factor receptor associated factor; TBK1, TANK-binding kinase 1; IKK, IκB kinase; IRF, interferon regulatory factor; NEMO, NF-κB essential modulator; IκB, NF-κB inhibitor; NF-κB, nuclear factor-kappaB; IFNAR, IFN-α/β receptor; TYK2, tyrosine kinase 2; JAK1, Janus kinase 1; STAT, signal transducer and activator of transcription; ISG, interferon-stimulated gene.

PDCoV infection observably upregulate genes associated with the RIG-I-like receptor signaling pathway, toll-like receptor signaling pathway and so on (44, 71). These pathways are responsible for sensing of the invading viral RNA and activating downstream signaling cascades to produce cytokines. PDCoV infection induces the expression of IFN-α, IFN-β, IFN-γ, IFN-λ1, IL-22, IL- 1β, IL-6, IL-12, TNF-α and IFN-λ1 *in vitro* or *in vivo* (2, 44, 48, 50, 64, 72). Coinfection with PDCoV and PEDV has a synergetic effect on the regulation of cytokines (73, 74). In the case of PDCoV-PEDV coinfection, the expressions of IFN-α, IL-6, IL-8, IL-12 and TNF-α are significantly up-regulated compared with single PDCoV infection. Meanwhile, PDCoV has evolved diversiform escape strategies to interfere with the host's innate immunity (Figure 1). PDCoV nsp5 inhibits IFN-β production by cleavage NF-κB essential modulator (NEMO) at the cleavage site of glutamine 231 (Q231) (75). This is similar to the mechanism by which porcine epidemic diarrhea virus (PEDV) nsp5 inhibits IFN-β production, implying that the same IFN antagonistic mechanism may be common to members of the *Coronaviridae* family (76). Moreover, PDCoV nsp5 antagonizes type I IFN signaling by cleaving signal transducer and activator of transcription 2 (STAT2) at two residues, Q685 and Q758, and the protease activity of nsp5 determines STAT2 cleavage (77). PDCoV nsp15 inhibits IFN-β production by destroying NF-κB activation via an endoribonuclease activity-independent mechanism, but does not antagonize interferon regulatory factor 3 (IRF3) activation (78). PDCoV NS6 interacts with RIG-I/MDA5, which in turn interferes with the binding of RIG-I/MDA5 to double-stranded RNA (dsRNA), resulting in the reduction of RLR-mediated IFN-β production (79). Whereas, Deng et al. removed NS6 from the infectious clone of PDCoV and found that the deletion of NS6 did not affect the ability of the infectious clone virus to inhibit the type I IFN responses, implying that NS6 does not act as an IFN antagonist during PDCoV infection (80). PDCoV NS7a competes with tumor necrosis factor receptor-associated factor 3 (TRAF3) and IRF3 for binding to IκB kinase ε (IKKε) by interacting with the kinase domain and the scaffold dimerization domain of IKKε simultaneously, forming an antagonistic effect on RLR-mediated IFN-β production (81). PDCoV N protein is also an IFN antagonist. It interacts with the helicase domain and the C-terminal domain of RIG-I, impairs dsRNA and protein activator of protein kinase R (PACT) binding to RIG-I, and ultimately antagonizes IFN-β production (82). The N-terminal region (1-246 aa) of N protein is critical for the interaction between N protein and RIG-I. Besides, N protein inhibits RIG-I K63-linked polyubiquitination by disrupting the binding of Riplet (an important activator for RIG-I by mediating the K63-linked polyubiquitination) to RIG-I, thus hampering the production of IFN-β (83). N protein also suppresses IRF7-induced type I interferon production via ubiquitin-proteasomal degradation pathway. It interacts with porcine IRF7 in a species-specific manner and promotes IRF7 degradation mostly through the K6, K11, and K29-linked polyubiquitination. Lysine 359 of IRF7 was a key site for N protein to induce IRF7 degradation (84). PDCoV infection also antagonizes IFN-λ1 production by decreasing the number of peroxisomes without altering the expression levels of IRF1 and mitochondrial antiviral-signaling (MAVS) (85). Peroxisome is the platform for MAVS to activate IRF1, and peroxisome-localized MAVS is associated with the production of IFN-λs.

## Current Anti-PDCoV Strategies

At present, there is little information about commercially available drugs or vaccines for PDCoV. Antiviral reagents mainly have two types of inhibition mechanisms: (i) targeting the virus themselves or (ii) targeting host factors. Virus-targeting antiviral agents can impede viral replication process or suppress the biological functions of viral proteins, mainly enzyme activities. PDCoV replication begins with the viral attachment and entry. After that, the virus uncoats and begins translation, transcription, RNA and protein synthesis, assembly, maturation and release. Disruption of any of these processes can impair viral replication. Host-targeting antiviral agents target host factors involved in PDCoV infection, regulating the function of the immune system or other cellular processes. Such agents have advantages in reducing viral resistance and managing viral emergencies.

Remdesivir is a monophosphoramidate prodrug of an adenosine analog that has efficient antiviral ability against a range of RNA viruses by targeting viral RNA dependent RNA polymerase, which is required for viral RNA synthesis. Treatment with remdesivir diminishes PDCoV replication in a dose-dependent manner with an EC_50_ value of 0.02 μM in Huh7 cells cultured in TPCK trypsin-containing and serum-free media (86). However, the inhibitory effects are not obvious in LLC-PK1 cells. This may be due to the lack of cellular processes required for the antiviral activity of remdesivir in LLC-PK1 cells. Lithium chloride (LC) and diammonium glycyrrhizinate (DG) exert anti-PDCoV activities at the early stage of PDCoV replication, and DG but not LC inhibits viral attachment (87). Furthermore, both drugs attenuated PDCoV-induced apoptosis in LLC-PK1 cells. Rhodanine derivative LJ001 possesses effective inhibitory ability at the replication stage of PDCoV life cycle in ST cells. It restricts the synthesis of viral RNA and protein, and reduces viral production (88). 25-hydroxycholesterol (25HC), the product of cholesterol oxidation by the enzyme CH25H, inhibits PDCoV infection by blocking PDCoV entry (70). Bile acids chenodeoxycholic acid and lithocholic acid suppress PDCoV replication at post-entry stages by inducing the production of IFN-λ3 and IFN-stimulated gene 15 (ISG15). Lithocholic acid functions through a G protein-coupled receptor-IFN-λ3-ISG15 signaling axis in IPEC-J2 cells (89). Melatonin and structural analogs, including indole, tryptamine and L-tryptophan possess antiviral activities against PDCoV. Further studies ascertain that melatonin exerts antiviral effects at the early stage of viral replication with no effect on viral attachment but an effect on viral entry (90). Our group found that ergosterol peroxide (EP) exhibits anti-PDCOV activities targeting viral attachment, viral entry, and the early and middle stages of PDCoV replication *in vitro*, and protects piglets from PDCoV infection to a large extent (2, 64, 66). Inhibition of PDCoV-induced p38 activation is one of the mechanisms by which EP inhibits viral replication. Furthermore, EP inactivates PDCoV infectivity directly. This inhibition occurs before the virus invades host cells, which is an important direction to develop antiviral drugs. EP also ameliorates PDCoV-induced apoptosis and autophagy, and displays immunomodulatory capacity due to its suppression of NF-κB during PDCoV infection. *In vivo* study demonstrates that EP also enhances tight junction protein expressions in the small intestine.

Strategies for PDCoV vaccine development include inactivated virus vaccines, plasmids expressing shRNAs, live-attenuated virus vaccines and virus-like particles (VLPs). An inactivated PDCoV vaccine was prepared by inactivating the 15^th^ generation virus of PDCoV strain NH with β-propiolactone containing aluminum hydroxide adjuvant at a 1:1 ratio, and pregnant sows were vaccinated at Houhai acupoint using a prime/boost strategy 20 and 40 days before delivery (91). The inactivated vaccine induced PDCoV S-specific IgG and neutralizing antibody (NA) responses in immunized sows and piglets from immunized sows, and exhibited 87.1% protective efficacy in the piglets which were orally challenged with PDCoV strain NH (10^5^ TCID_50/_piglet) at 5 days post-farrowing. Gao et al. developed inactivated PDCoV vaccines by mixing binary ethyleneimine-inactivated cell culture-adapted CH/XJYN/2016 P30 with 206 adjuvant or Imject™ Alum Adjuvant (92). Gu et al. found that short hairpin RNAs targeting M and N genes diminished PDCoV replication in ST cells and proposed that RNA interference (RNAi) is a promising new strategy against PDCoV infection (93). Hereafter, a double-shRNA-expression plasmid specific to N gene, named pSil-double-shRNA-N1, were designed by Gu et al. (94). Zhang et al. replaced the NS6 gene of a full-length infectious cDNA clone of PDCoV with a green fluorescent protein (GFP) to generate rPDCoV-ΔNS6-GFP (95). rPDCoV-ΔNS6-GFP exhibited a decrease in viral yield *in vitro* and *in vivo*, and rPDCoV-ΔNS6-GFP infected piglets showed hardly detectable clinical symptoms or intestinal lesions. These results indicate that NS6 protein is an important virulence factor of PDCoV, providing a potential candidate for the development of live-attenuated virus vaccine. VLPs are non-genetic multimeric nanoparticles synthesized through *in vitro* or *in vivo* self-assembly of one or more viral structural proteins. Guo et al. synthesized chimeric VLP vaccines of the phage Qbeta coat protein presenting the universal epitope of coronavirus (96). The chimeric VLP vaccines induced NA responses against MHV, PEDV and PDCoV in mice.

## Conclusion

PDCoV is a novel swine enteropathogenic coronavirus with worldwide distribution, causing great economic losses to pig industry. PDCoV can also infect calves, chickens, turkeys, mice, and even humans, emphasizing its possible ability to cross interspecies barriers. Its rapid dissemination and potential cross-species transmissibility impose a huge burden on animal and human health safety. In the past 20 years, as several coronaviruses have crossed the species barrier to infect humans and new coronaviruses have emerged, serious diseases have broken out. Therefore, global vigilance against coronavirus-related diseases needs to be strengthened. Grasping the epidemical situation, pathogenesis and immune escape strategies of PDCoV is essential for intervention in PDCoV infection, and it is even possible to discover some common signaling pathways and key molecules of coronaviruses. In addition, this review summarizes the current research status of drugs and vaccines against PDCoV, providing valuable clues for the screening of PDCoV drugs and the design of new prevention strategies.

## Author Contributions

The author confirms being the sole contributor of this work and has approved it for publication.

## Conflict of Interest

The author declares that the research was conducted in the absence of any commercial or financial relationships that could be construed as a potential conflict of interest.

## Publisher's Note

All claims expressed in this article are solely those of the authors and do not necessarily represent those of their affiliated organizations, or those of the publisher, the editors and the reviewers. Any product that may be evaluated in this article, or claim that may be made by its manufacturer, is not guaranteed or endorsed by the publisher.

## References

[1] Jung K Hu H Eyerly B Lu Z Chepngeno J Saif LJ. Pathogenicity of 2 porcine deltacoronavirus strains in gnotobiotic pigs. Emerg Infect Dis. (2015) 21:650–4. 10.3201/eid2104.141859 25811229 PMC4378491

[2] Duan C Wang J Liu Y Zhang J Si J Hao Z. Antiviral effects of ergosterol peroxide in a pig model of porcine deltacoronavirus (PDCoV) infection involves modulation of apoptosis and tight junction in the small intestine. Vet Res. (2021) 52:86. 10.1186/s13567-021-00955-5 34127062 PMC8201433

[3] Wang L Byrum B Zhang Y. Detection and genetic characterization of deltacoronavirus in pigs, Ohio, USA, 2014. Emerg Infect Dis. (2014) 20:1227–30. 10.3201/eid2007.140296 24964136 PMC4073853

[4] Li G Chen Q Harmon KM Yoon KJ Schwartz KJ Hoogland MJ. Full-length genome sequence of porcine deltacoronavirus strain USA/IA/2014/8734. Genome Announc. (2014) 2:e00278–14. 10.1128/genomeA.00278-14 24723718 PMC3983307

[5] Choi S Lee C. Functional characterization and proteomic analysis of porcine deltacoronavirus accessory protein NS7. J Microbiol Biotechnol. (2019) 29:1817–29. 10.4014/jmb.1908.08013 31546302

[6] Fang P Fang L Liu X Hong Y Wang Y Dong N. Identification and subcellular localization of porcine deltacoronavirus accessory protein NS6. Virology. (2016) 499:170-7. 10.1016/j.virol.2016.09.015 27661736 PMC7111631

[7] Fang P Fang L Hong Y Liu X Dong N Ma P. Discovery of a novel accessory protein NS7a encoded by porcine deltacoronavirus. J Gen Virol. (2017) 98:173–8. 10.1099/jgv.0.000690 27995863 PMC7079566

[8] Qin P Luo WT Su Q Zhao P Zhang P Wang B. The porcine deltacoronavirus accessory protein NS6 is expressed in vivo and incorporated into virions. Virology. (2021) 556:1–8. 10.1016/j.virol.2021.01.011 33515858 PMC7825830

[9] Woo PC Lau SK Lam CS Lau CC Tsang AK Lau JH. Discovery of seven novel mammalian and avian coronaviruses in the genus deltacoronavirus supports bat coronaviruses as the gene source of alphacoronavirus and betacoronavirus and avian coronaviruses as the gene source of gammacoronavirus and deltacoronavirus. J Virol. (2012) 86:3995–4008. 10.1128/JVI.06540-11 22278237 PMC3302495

[10] Jang G Lee KK Kim SH Lee C. Prevalence, complete genome sequencing and phylogenetic analysis of porcine deltacoronavirus in South Korea, 2014–2016. Transbound Emerg Dis. (2017) 64:1364–70. 10.1111/tbed.12690 28758347 PMC7169712

[11] Suzuki T Shibahara T Imai N Yamamoto T Ohashi S. Genetic characterization and pathogenicity of Japanese porcine deltacoronavirus. Infect Genet Evol. (2018) 61:176–82. 10.1016/j.meegid.2018.03.030 29621617 PMC7172274

[12] Dong N Fang L Yang H Liu H Du T Fang P. Isolation, genomic characterization, and pathogenicity of a Chinese porcine deltacoronavirus strain CHN-HN-2014. Vet Microbiol. (2016) 196:98–106. 10.1016/j.vetmic.2016.10.022 27939164 PMC7117368

[13] Jung K Hu H Saif LJ. Calves are susceptible to infection with the newly emerged porcine deltacoronavirus, but not with the swine enteric alphacoronavirus, porcine epidemic diarrhea virus. Arch Virol. (2017) 162:2357–62. 10.1007/s00705-017-3351-z 28374120 PMC7086908

[14] Liang Q Zhang H Li B Ding Q Wang Y Gao W. Susceptibility of chickens to porcine deltacoronavirus infection. Viruses. (2019) 11:573. 10.3390/v11060573 31234434 PMC6631122

[15] Boley PA Alhamo MA Lossie G Yadav KK Vasquez-Lee M Saif LJ. Porcine deltacoronavirus infection and transmission in poultry, United States^1^. Emerg Infect Dis. (2020) 26:255–65. 10.3201/eid2602.190346 31961296 PMC6986833

[16] Liu Y Wang B Liang QZ Shi FS Ji CM Yang XL. The roles of two major domains of the porcine deltacoronavirus spike subunit 1 in receptor binding and neutralization. J Virol. (2021) 95:e0111821. 10.1128/JVI.01118-21 34549985 PMC8610578

[17] Lednicky JA Tagliamonte MS White SK Elbadry MA Alam MM Stephenson CJ. Independent infections of porcine deltacoronavirus among Haitian children. Nature. (2021) 600:133–7. 10.1038/s41586-021-04111-z 34789872 PMC8636265

[18] Wang L Byrum B Zhang Y. Porcine coronavirus HKU15 detected in 9 US states, 2014. Emerg Infect Dis. (2014) 20:1594–5. 10.3201/eid2009.140756 25153521 PMC4178395

[19] Ajayi T Dara R Misener M Pasma T Moser L Poljak Z. Herd-level prevalence and incidence of porcine epidemic diarrhoea virus (PEDV) and porcine deltacoronavirus (PDCoV) in swine herds in Ontario, Canada. Transbound Emerg Dis. (2018) 65:1197–207. 10.1111/tbed.12858 29607611 PMC7169835

[20] Song D Zhou X Peng Q Chen Y Zhang F Huang T. Newly emerged porcine deltacoronavirus associated with diarrhoea in swine in China: identification, prevalence and full-length genome sequence analysis. Transbound Emerg Dis. (2015) 62:575–80. 10.1111/tbed.12399 26250097 PMC7169704

[21] Saeng-Chuto K Lorsirigool A Temeeyasen G Vui DT Stott CJ Madapong A. Different lineage of porcine deltacoronavirus in Thailand, Vietnam and Lao PDR in 2015. Transbound Emerg Dis. (2017) 64:3–10. 10.1111/tbed.12585 27718337 PMC7169859

[22] Perez-Rivera C Ramirez-Mendoza H Mendoza-Elvira S Segura-Velazquez R Sanchez-Betancourt JI. First report and phylogenetic analysis of porcine deltacoronavirus in Mexico. Transbound Emerg Dis. (2019) 66:1436–41. 10.1111/tbed.13193 30941894 PMC7168549

[23] Vicente-Huaman J Gómez-Quispe OE. Evaluation of a porcine deltacoronavirus eradication program in a full-cycle pig farm in Peru. J Adv Vet Anim Res. (2021) 8:300–6. 10.5455/javar.2021.h515 34395601 PMC8280984

[24] Thachil A Gerber PF Xiao CT Huang YW Opriessnig T. Development and application of an ELISA for the detection of porcine deltacoronavirus IgG antibodies. PLoS ONE. (2015) 10:e0124363. 10.1371/journal.pone.0124363 25881086 PMC4399883

[25] Dong N Fang L Zeng S Sun Q Chen H Xiao S. Porcine deltacoronavirus in mainland China. Emerg Infect Dis. (2015) 21:2254–5. 10.3201/eid2112.150283 26584185 PMC4672429

[26] He WT Ji X He W Dellicour S Wang S Li G. Genomic epidemiology, evolution, and transmission dynamics of porcine deltacoronavirus. Mol Biol Evol. (2020) 37:2641–54. 10.1093/molbev/msaa117 32407507 PMC7454817

[27] Zhang Y Cheng Y Xing G Yu J Liao A Du L. Detection and spike gene characterization in porcine deltacoronavirus in China during 2016–2018. Infect Genet Evol. (2019) 73:151–8. 10.1016/j.meegid.2019.04.023 31026605 PMC7106087

[28] Marthaler D Raymond L Jiang Y Collins J Rossow K Rovira A. Rapid detection, complete genome sequencing, and phylogenetic analysis of porcine deltacoronavirus. Emerg Infect Dis. (2014) 20:1347–50. 10.3201/eid2008.140526 25075556 PMC4111195

[29] Zhai SL Wei WK Li XP Wen XH Zhou X Zhang H. Occurrence and sequence analysis of porcine deltacoronaviruses in southern China. Virol J. (2016) 13:136. 10.1186/s12985-016-0591-6 27496131 PMC4974758

[30] Mai K Feng J Chen G Li D Zhou L Bai Y. The detection and phylogenetic analysis of porcine deltacoronavirus from Guangdong province in southern China. Transbound Emerg Dis. (2018) 65:166–73. 10.1111/tbed.12644 28345292 PMC7169752

[31] Hsu TH Liu HP Chin CY Wang C Zhu WZ Wu BL. Detection, sequence analysis, and antibody prevalence of porcine deltacoronavirus in Taiwan. Arch Virol. (2018) 163:3113–7. 10.1007/s00705-018-3964-x 30051342 PMC7086614

[32] Zhang H Liang Q Li B Cui X Wei X Ding Q. Prevalence, phylogenetic and evolutionary analysis of porcine deltacoronavirus in Henan province, China. Prev Vet Med. (2019) 166:8–15. 10.1016/j.prevetmed.2019.02.017 30935509 PMC7114282

[33] Zhang F Luo S Gu J Li Z Li K Yuan W. Prevalence and phylogenetic analysis of porcine diarrhea associated viruses in southern China from 2012 to 2018. BMC Vet Res. (2019) 15:470. 10.1186/s12917-019-2212-2 31881873 PMC6935106

[34] Saeng-Chuto K Jermsutjarit P Stott CJ Vui DT Tantituvanont A Nilubol D. Retrospective study, full-length genome characterization and evaluation of viral infectivity and pathogenicity of chimeric porcine deltacoronavirus detected in Vietnam. Transbound Emerg Dis. (2020) 67:183–98. 10.1111/tbed.13339 31469947 PMC7168546

[35] Feng Y Xu Z Zhu L. Prevalence and phylogenetic analysis of porcine deltacoronavirus in Sichuan province, China. Arch Virol. (2020) 165:2883–9. 10.1007/s00705-020-04796-z 32892248 PMC7474797

[36] Hu H Jung K Vlasova AN Saif LJ. Experimental infection of gnotobiotic pigs with the cell-culture-adapted porcine deltacoronavirus strain OH-FD22. Arch Virol. (2016) 161:3421–34. 10.1007/s00705-016-3056-8 27619798 PMC7087098

[37] Janetanakit T Lumyai M Bunpapong N Boonyapisitsopa S Chaiyawong S Nonthabenjawan N. Porcine deltacoronavirus, Thailand, 2015. Emerg Infect Dis. (2016) 22:757–9. 10.3201/eid2204.151852 26982324 PMC4806967

[38] Le VP Song S An BH Park GN Pham NT Le DQ. A novel strain of porcine deltacoronavirus in Vietnam. Arch Virol. (2018) 163:203–7. 10.1007/s00705-017-3594-8 29022111 PMC7087264

[39] Vitosh-Sillman S Loy JD Brodersen B Kelling C Doster A Topliff C. Experimental infection of conventional nursing pigs and their dams with porcine deltacoronavirus. J Vet Diagn Invest. (2016) 28:486–97. 10.1177/1040638716654200 27578872

[40] Ma Y Zhang Y Liang X Lou F Oglesbee M Krakowka S. Origin, evolution, and virulence of porcine deltacoronaviruses in the United States. mBio. (2015) 6:e00064. 10.1128/mBio.00064-15 25759498 PMC4453528

[41] Chen Q Gauger P Stafne M Thomas J Arruda P Burrough E. Pathogenicity and pathogenesis of a United States porcine deltacoronavirus cell culture isolate in 5-day-old neonatal piglets. Virology. (2015) 482:51–9. 10.1016/j.virol.2015.03.024 25817405 PMC7111688

[42] Wu JL Mai KJ Li D Wu RT Wu ZX Tang XY. Expression profile analysis of 5-day-old neonatal piglets infected with porcine deltacoronavirus. BMC Vet Res. (2019) 15:117. 10.1186/s12917-019-1848-2 30992015 PMC6469071

[43] Xu Z Zhong H Zhou Q Du Y Chen L Zhang Y. A highly pathogenic strain of porcine deltacoronavirus caused watery diarrhea in newborn piglets. Virol Sin. (2018) 33:131–41. 10.1007/s12250-018-0003-8 29569144 PMC6178105

[44] Xu Z Zhong H Huang S Zhou Q Du Y Chen L. Porcine deltacoronavirus induces TLR3, IL-12, IFN-α, IFN-β and PKR mRNA expression in infected Peyer's patches *in vivo*. Vet Microbiol. (2019) 228:226–33. 10.1016/j.vetmic.2018.12.012 30593372 PMC7117130

[45] Wang H Qin Y Zhao W Yuan T Yang C Mi X. Genetic characteristics and pathogenicity of a novel porcine deltacoronavirus southeast Asia-like strain found in China. Front Vet Sci. (2021) 8:701612. 10.3389/fvets.2021.701612 34336982 PMC8322666

[46] Homwong N Jarvis MC Lam HC Diaz A Rovira A Nelson M. Characterization and evolution of porcine deltacoronavirus in the United States. Prev Vet Med. (2016) 123:168–74. 10.1016/j.prevetmed.2015.11.001 26611652 PMC7114263

[47] Yang YL Meng F Qin P Herrler G Huang YW Tang YD. Trypsin promotes porcine deltacoronavirus mediating cell-to-cell fusion in a cell type-dependent manner. Emerg Microbes Infect. (2020) 9:457–68. 10.1080/22221751.2020.1730245 32090689 PMC7054919

[48] Hu H Jung K Vlasova AN Chepngeno J Lu Z Wang Q. Isolation and characterization of porcine deltacoronavirus from pigs with diarrhea in the United States. J Clin Microbiol. (2015) 53:1537–48. 10.1128/JCM.00031-15 25740769 PMC4400786

[49] Qin P Du EZ Luo WT Yang YL Zhang YQ Wang B. Characteristics of the life cycle of porcine deltacoronavirus (PDCoV) *in vitro*: replication kinetics, cellular ultrastructure and virion morphology, and evidence of inducing autophagy. Viruses. (2019) 11:455. 10.3390/v11050455 31109068 PMC6563515

[50] Yin L Chen J Li L Guo S Xue M Zhang J. Aminopeptidase N expression, not interferon responses, determines the intestinal segmental tropism of porcine deltacoronavirus. J Virol. (2020) 94:e00480–20. 10.1128/JVI.00480-20 32376622 PMC7343211

[51] Xiong X Tortorici MA Snijder J Yoshioka C Walls AC Li W. Glycan shield and fusion activation of a deltacoronavirus spike glycoprotein fine-tuned for enteric infections. J Virol. (2018) 92:e01628–17. 10.1128/JVI.01628-17 29093093 PMC5790929

[52] Shang J Zheng Y Yang Y Liu C Geng Q Tai W. Cryo-electron microscopy structure of porcine deltacoronavirus spike protein in the prefusion state. J Virol. (2018) 92:e01556–17. 10.1128/JVI.01556-17 29070693 PMC5790952

[53] Wang B Liu Y Ji CM Yang YL Liang QZ Zhao P. Porcine deltacoronavirus engages the transmissible gastroenteritis virus functional receptor porcine aminopeptidase N for infectious cellular entry. J Virol. (2018) 92:e00318–18. 10.1128/JVI.00318-18 29618640 PMC5974500

[54] Li W Hulswit RJ Kenney SP Widjaja I Jung K Alhamo MA. Broad receptor engagement of an emerging global coronavirus may potentiate its diverse cross-species transmissibility. Proc Natl Acad Sci USA. (2018) 115:E5135–43. 10.1073/pnas.1802879115 29760102 PMC5984533

[55] Xu K Zhou Y Mu Y Liu Z Hou S Xiong Y. CD163 and pAPN double-knockout pigs are resistant to PRRSV and TGEV and exhibit decreased susceptibility to PDCoV while maintaining normal production performance. ELife. (2020) 9:e57132. 10.7554/eLife.57132.sa2 32876563 PMC7467724

[56] Zhu X Liu S Wang X Luo Z Shi Y Wang D. Contribution of porcine aminopeptidase N to porcine deltacoronavirus infection. Emerg Microbes Infect. (2018) 7:65. 10.1038/s41426-018-0068-3 29636467 PMC5893578

[57] Stoian A Rowland RR Petrovan V Sheahan M Samuel MS Whitworth KM. The use of cells from ANPEP knockout pigs to evaluate the role of aminopeptidase N (APN) as a receptor for porcine deltacoronavirus (PDCoV). Virology. (2020) 541:136–40. 10.1016/j.virol.2019.12.007 32056711 PMC7112016

[58] Delmas B Gelfi J L'Haridon R Vogel LK Sjostrom H Noren O. Aminopeptidase N is a major receptor for the entero-pathogenic coronavirus TGEV. Nature. (1992) 357:417–20. 10.1038/357417a0 1350661 PMC7095137

[59] Zhang J Chen J Shi D Shi H Zhang X Liu J. Porcine deltacoronavirus enters cells via two pathways: a protease-mediated one at the cell surface and another facilitated by cathepsins in the endosome. J Biol Chem. (2019) 294:9830–43. 10.1074/jbc.RA119.007779 31068417 PMC6597833

[60] Fang P Zhang J Zhang H Xia S Ren J Tian L. Porcine deltacoronavirus enters porcine IPI-2I intestinal epithelial cells via macropinocytosis and clathrin-mediated endocytosis dependent on pH and dynamin. J Virol. (2021) 95:e0134521. 10.1128/JVI.01345-21 34586858 PMC8610596

[61] Yang YL Liu J Wang TY Chen M Wang G Yang YB. Aminopeptidase N is an entry co-factor triggering porcine deltacoronavirus entry via an endocytotic pathway. J Virol. (2021) 95:e0094421. 10.1128/JVI.00944-21 34406863 PMC8513460

[62] Jeon JH Lee C. Cholesterol is important for the entry process of porcine deltacoronavirus. Arch Virol. (2018) 163:3119–24. 10.1007/s00705-018-3967-7 30051343 PMC7087128

[63] Jeon JH Lee YJ Lee C. Porcine deltacoronavirus activates the Raf/MEK/ERK pathway to promote its replication. Virus Res. (2020) 283:197961. 10.1016/j.virusres.2020.197961 32283129 PMC7194644

[64] Duan C Ge X Wang J Wei Z Feng WH Wang J. Ergosterol peroxide exhibits antiviral and immunomodulatory abilities against porcine deltacoronavirus (PDCoV) via suppression of NF-κB and p38/MAPK signaling pathways *in vitro*. Int Immunopharmacol. (2021) 93:107317. 10.1016/j.intimp.2020.107317 33493866 PMC9412180

[65] Jeon JH Lee C. Stress-activated protein kinases are involved in the replication of porcine deltacoronavirus. Virology. (2021) 559:196–209. 10.1016/j.virol.2021.04.007 33964685

[66] Duan C Liu Y Hao Z Wang J. Ergosterol peroxide suppresses porcine deltacoronavirus (PDCoV)-induced autophagy to inhibit virus replication via p38 signaling pathway. Vet Microbiol. (2021) 257:109068. 10.1016/j.vetmic.2021.109068 33894664 PMC8035807

[67] Lee YJ Lee C. Porcine deltacoronavirus induces caspase-dependent apoptosis through activation of the cytochrome c-mediated intrinsic mitochondrial pathway. Virus Res. (2018) 253:112–23. 10.1016/j.virusres.2018.06.008 29940190 PMC7114866

[68] Jung K Hu H Saif LJ. Porcine deltacoronavirus induces apoptosis in swine testicular and LLC porcine kidney cell lines *in vitro* but not in infected intestinal enterocytes *in vivo*. Vet Microbiol. (2016) 182:57–63. 10.1016/j.vetmic.2015.10.022 26711029 PMC7117480

[69] Bai D Fang L Xia S Ke W Wang J Wu X. Porcine deltacoronavirus (PDCoV) modulates calcium influx to favor viral replication. Virology. (2020) 539:38–48. 10.1016/j.virol.2019.10.011 31670218 PMC7112098

[70] Ke W Wu X Fang P Zhou Y Fang L Xiao S. Cholesterol 25-hydroxylase suppresses porcine deltacoronavirus infection by inhibiting viral entry. Virus Res. (2021) 295:198306. 10.1016/j.virusres.2021.198306 33476696 PMC7833861

[71] Zhou X Zhou L Ge X Guo X Han J Zhang Y. Quantitative proteomic analysis of porcine intestinal epithelial cells infected with porcine deltacoronavirus using iTRAQ-coupled LC-MS/MS. J Proteome Res. (2020) 19:4470–85. 10.1021/acs.jproteome.0c00592 33045833

[72] Zhao D Gao X Zhou P Zhang L Zhang Y Wang Y. Evaluation of the immune response in conventionally weaned pigs infected with porcine deltacoronavirus. Arch Virol. (2020) 165:1653–8. 10.1007/s00705-020-04590-x 32399787 PMC7215125

[73] Zhang H Han F Shu X Li Q Ding Q Hao C. Co-infection of porcine epidemic diarrhoea virus and porcine deltacoronavirus enhances the disease severity in piglets. Transbound Emerg Dis. (2021). 10.1111/tbed.14144. [Epub ahead of print]. 33960702

[74] Saeng-Chuto K Madapong A Kaeoket K Piñeyro PE Tantituvanont A Nilubol D. Coinfection of porcine deltacoronavirus and porcine epidemic diarrhea virus increases disease severity, cell trophism and earlier upregulation of IFN-α and IL12. Sci Rep. (2021) 11:3040. 10.1038/s41598-021-82738-8 33542409 PMC7862360

[75] Zhu X Fang L Wang D Yang Y Chen J Ye X. Porcine deltacoronavirus nsp5 inhibits interferon-β production through the cleavage of NEMO. Virology. (2017) 502:33–8. 10.1016/j.virol.2016.12.005 27984784 PMC7111669

[76] Wang D Fang L Shi Y Zhang H Gao L Peng G. Porcine epidemic diarrhea virus 3C-like protease regulates its interferon antagonism by cleaving NEMO. J Virol. (2016) 90:2090–101. 10.1128/JVI.02514-15 26656704 PMC4733996

[77] Zhu X Wang D Zhou J Pan T Chen J Yang Y. Porcine deltacoronavirus nsp5 antagonizes type I interferon signaling by cleaving STAT2. J Virol. (2017) 91:e00003–17. 10.1128/JVI.00003-17 28250121 PMC5411617

[78] Liu X Fang P Fang L Hong Y Zhu X Wang D. Porcine deltacoronavirus nsp15 antagonizes interferon-β production independently of its endoribonuclease activity. Mol Immunol. (2019) 114:100–7. 10.1016/j.molimm.2019.07.003 31351410 PMC7112593

[79] Fang P Fang L Ren J Hong Y Liu X Zhao Y. Porcine deltacoronavirus accessory protein NS6 antagonizes interferon beta production by interfering with the binding of RIG-I/MDA5 to double-stranded RNA. J Virol. (2018) 92:e00712–18. 10.1128/JVI.00712-18 29769346 PMC6052322

[80] Deng X Buckley AC Pillatzki A Lager KM Baker SC Faaberg KS. Development and utilization of an infectious clone for porcine deltacoronavirus strain USA/IL/2014/026. Virology. (2021) 553:35–45. 10.1016/j.virol.2020.11.002 33220618 PMC7664480

[81] Fang P Fang L Xia S Ren J Zhang J Bai D. Porcine deltacoronavirus accessory protein NS7a antagonizes IFN-β production by competing with TRAF3 and IRF3 for binding to IKKε. Front Cell Infect Microbiol. (2020) 10:257. 10.3389/fcimb.2020.00257 32656094 PMC7326017

[82] Chen J Fang P Wang M Peng Q Ren J Wang D. Porcine deltacoronavirus nucleocapsid protein antagonizes IFN-β production by impairing dsRNA and PACT binding to RIG-I. Virus Genes. (2019) 55:520–31. 10.1007/s11262-019-01673-z 31129785 PMC7088841

[83] Ji L Li S Zhu W Ma J Sun J Wang H. Porcine deltacoronavirus nucleocapsid protein suppressed IFN-β production by interfering porcine RIG-I dsRNA-binding and K63-linked polyubiquitination. Front Immunol. (2019) 10:1024. 10.3389/fimmu.2019.01024 31143181 PMC6521028

[84] Ji L Wang N Ma J Cheng Y Wang H Sun J. Porcine deltacoronavirus nucleocapsid protein species-specifically suppressed IRF7-induced type I interferon production via ubiquitin-proteasomal degradation pathway. Vet Microbiol. (2020) 250:108853. 10.1016/j.vetmic.2020.108853 32992291 PMC7834071

[85] Liu S Fang P Ke W Wang J Wang X Xiao S. Porcine deltacoronavirus (PDCoV) infection antagonizes interferon-λ1 production. Vet Microbiol. (2020) 247:108785. 10.1016/j.vetmic.2020.108785 32768229 PMC7331541

[86] Brown AJ Won JJ Graham RL Dinnon KH 3rd Sims AC Feng JY. Broad spectrum antiviral remdesivir inhibits human endemic and zoonotic deltacoronaviruses with a highly divergent RNA dependent RNA polymerase. Antiviral Res. (2019) 169:104541. 10.1016/j.antiviral.2019.104541 31233808 PMC6699884

[87] Zhai X Wang S Zhu M He W Pan Z Su S. Antiviral effect of lithium chloride and diammonium glycyrrhizinate on porcine deltacoronavirus *in vitro*. Pathogens. (2019) 8:144. 10.3390/pathogens8030144 31505777 PMC6789623

[88] Zhang Y Xia L Yuan Y Li Q Han L Yang G. Rhodanine derivative LJ001 inhibits TGEV and PDCoV replication *in vitro*. Virus Res. (2020) 289:198167. 10.1016/j.virusres.2020.198167 32956749 PMC7501054

[89] Kong F Niu X Liu M Wang Q. Bile acids LCA and CDCA inhibited porcine deltacoronavirus replication *in vitro*. Vet Microbiol. (2021) 257:109097. 10.1016/j.vetmic.2021.109097 33933854

[90] Zhai X Wang N Jiao H Zhang J Li C Ren W. Melatonin and other indoles show antiviral activities against swine coronaviruses *in vitro* at pharmacological concentrations. J Pineal Res. (2021) 71:e12754. 10.1111/jpi.12754 34139040

[91] Zhang J Chen J Liu Y Da S Shi H Zhang X. Pathogenicity of porcine deltacoronavirus (PDCoV) strain NH and immunization of pregnant sows with an inactivated PDCoV vaccine protects 5-day-old neonatal piglets from virulent challenge. Transbound Emerg Dis. (2020) 67:572–83. 10.1111/tbed.13369 31541590 PMC7168751

[92] Gao X Zhao D Zhou P Zhang L Li M Li W. Characterization, pathogenicity and protective efficacy of a cell culture-derived porcine deltacoronavirus. Virus Res. (2020) 282:197955. 10.1016/j.virusres.2020.197955 32247757 PMC7125813

[93] Gu WY Li Y Liu BJ Wang J Yuan GF Chen SJ. Short hairpin RNAs targeting M and N genes reduce replication of porcine deltacoronavirus in ST cells. Virus Genes. (2019) 55:795–801. 10.1007/s11262-019-01701-y 31463771 PMC7088929

[94] Gu J Li H Bi Z Li K Li Z Song D. Plasmids expressing shRNAs specific to the nucleocapsid gene inhibit the replication of porcinedeltacoronavirus *in vivo*. Animals. (2021) 11:1216. 10.3390/ani11051216 33922444 PMC8145914

[95] Zhang M Li W Zhou P Liu D Luo R Jongkaewwattana A. Genetic manipulation of porcine deltacoronavirus reveals insights into NS6 and NS7 functions: a novel strategy for vaccine design. Emerg Microbes Infect. (2020) 9:20–31. 10.1080/22221751.2019.1701391 31859605 PMC6968670

[96] Guo Y Guo R Ma Y Chang W Ming S Yang G. Chimeric virus-like particles of universal antigen epitopes of coronavirus and phage Qβ coat protein trigger the production of neutralizing antibodies. Curr Top Med Chem. (2021) 21:1235–50. 10.2174/1568026621666210618145411 34145995

